# Quantitative Proteomic Analysis Identifies MAPK15 as a Potential Regulator of Radioresistance in Nasopharyngeal Carcinoma Cells

**DOI:** 10.3389/fonc.2018.00548

**Published:** 2018-11-22

**Authors:** Zhanzhan Li, Na Li, Liangfang Shen, Jun Fu

**Affiliations:** Department of Oncology, Xiangya Hospital, Central South University Changsha, China

**Keywords:** nasopharyngeal carcinoma, radioresistance, radiosensitivity, MAPK15, quantitative proteomics

## Abstract

Since resistance to radiotherapy remains refractory for the clinical management of nasopharyngeal cancer (NPC), further understanding the mechanisms of radioresistance is necessary in order to develop more effective NPC treatment and improve prognosis. In this study, an integrated quantitative proteomic approach involving tandem mass tag labeling and liquid chromatograph-mass spectrometer was used to identify proteins potentially responsible for the radioresistance of NPC. The differential radiosensitivity in NPC model cells was examined through clonogenic survival assay, CCK-8 viability assay, and BrdU incorporation analysis. Apoptosis of NPC cells after exposure to irradiation was detected using caspase-3 colorimetric assay. Intracellular reactive oxygen species (ROS) was detected by a dichlorofluorescin diacetate fluorescent probe. In total, 5,946 protein groups were identified, among which 5,185 proteins were quantified. KEGG pathway analysis and protein-protein interaction enrichment analysis revealed robust activation of multiple biological processes/pathways in radioresistant CNE2-IR cells. Knockdown of MAPK15, one up-regulated protein kinase in CNE2-IR cells, significantly impaired clonogenic survival, decreased cell viability and increased cell apoptosis following exposure to irradiation, while over-expression of MAPK15 promoted cell survival, induced radioresistance and reduced apoptosis in NPC cell lines CNE1, CNE2, and HONE1. MAPK15 might regulate radioresistance through attenuating ROS accumulation and promoting DNA damage repair after exposure to irradiation in NPC cells. Quantitative proteomic analysis revealed enormous metabolic processes/signaling networks were potentially involved in the radioresistance of NPC cells. MAPK15 might be a novel potential regulator of radioresistance in NPC cells, and targeting MAPK15 might be useful in sensitizing NPC cells to radiotherapy.

## Introduction

Nasopharyngeal carcinoma (NPC) is one of the most prevalent cancers in South China and Southeast Asia. Epidemiologic studies show the incidence of NPC is tightly linked to the Epstein-Barr virus (EBV) infection. The primary treatment for NPC is radiotherapy (1). Although better radiotherapy techniques and the more accurate tumor localization based on computed tomography have contributed to the improved local control, the local recurrence and distant metastasis of NPC are still the major causes of deaths and morbidity in advanced stages (2). Since a common cause of local recurrence and poor survival in NPC is radioresistance (3), understanding the mechanisms of radioresistance is necessary in order to develop more specific NPC treatment for and improve prognosis.

Although a fraction of genes such as elements of cell cycle control, apoptosis/anti-apoptosis, and DNA repair play key roles in ionizing radiation-induced cell damage, the intrinsic radioresistance of NPC remains poorly understood (4–6). Protein kinases, as key regulators of cell functions, are one of the largest and most functionally diverse gene families. Protein kinases direct the activity, localization and overall function of many proteins by adding phosphate groups to substrate proteins, and orchestrate the activity of almost all cellular processes. Emerging protein kinases are important in carcinogenesis and are being prioritized as drug targets. In this study, an integrated quantitative proteomic approach involving tandem mass tag (TMT) labeling and liquid chromatograph-tandem-mass spectrometer (LC-MS/MS) was used to identify potential proteins responsible for the radioresistance of NPC (7, 8). Comparative proteomics show numerous proteins involved in important bioprocesses are differentially altered in CNE2 and its radioresistant subline CNE2-IR. Mitogen-activated protein kinase 15 (MAPK15), one of the up-regulated protein kinases in CNE2-IR cells, was selected for further investigation. Our data suggest MAPK15 might be an important regulator of radioresistance in NPC cells, which may warrant MAPK15 as a potential therapeutic target in future investigations.

## Methods

### Reagents and cell lines

Antibodies against MAPK15, Src family tyrosine kinase (FYN), inhibitor of nuclear factor kappa B kinase subunit bet (IKBKB), mitogen-activated protein kinase 6 (MAP2K6), cyclin-dependent kinase 4 (CDK4), γ-H2AX, and β-actin were obtained from Abcam (Cambridge, USA). The reactive oxygen species (ROS) probe 2′,7′-dichlorodihydrofluorescein diacetate (DCFDA) was purchased from Thermo Fisher Scientific (Grand Island, New York, USA). Human NPC cell line CNE2 and its radioresistant subline CNE2-IR were well-characterized and widely used cell line models for the mechanistic study of radioresistance in NPC. To compare with previous study, we selected human NPC cell lines CNE2 and CNE2-IR kindly provided by Dr. Zhiqiang Xiao (Key Laboratory of Cancer Proteomics of Chinese Ministry of Health, Central South University, Changsha, China) (9, 10) who have reported some results. These NPC cell lines were cultured in Dulbecco's modified Eagle's medium supplemented with 10% fetal bovine serum (Hyclone, Logan, UT), 4 mM glutamine, 100 U/mL penicillin, and 100 μg/mL streptomycin.

### Protein extraction, trypsin digestion, and TMT labeling

CNE2 and CNE2-IR cell lines each with two biological replicates were used for quantitative proteomic analysis. NPC cells were ice-sonicated three times using a high-intensity ultrasonic processor (Scientz, Ningbo, China) in a lysis buffer (8 M urea, 2 mM ethylene diamine tetraacetic acid (EDTA), 10 mM dithiothreitol (DTT), 1% phosphatase inhibitor cocktail, and 1% protease inhibitor cocktail). The remaining debris was removed by centrifugation at 20,000 × g and 4°C for 10 min. Then the proteins were precipitated with 15% cold trichloroacetic acid for 2 h at −20°C. After centrifugation at 4°C for 10 min, the supernatant was discarded and the remaining precipitate was washed with cold acetone for three times. The proteins were redissolved in a buffer (8 M urea, 100 mM TEAB, pH 8.0) and the protein concentration was detected with 2-D Quant kit according to the manufacturer's instructions. For digestion, the protein solution was reduced with 10 mM DTT for 1 h at 37°C and alkylated with 20 mM IAA for 45 min at room temperature in darkness. For trypsin digestion, the protein solution was diluted by adding 100 mM TEAB until the urea concentration was < 2 M. Finally, trypsin was added at 1:50 and 1:100 trypsin-to-protein mass ratio for the first digestion overnight and a second 4 h-digestion, respectively.

After trypsin digestion, peptides were desalted by an Strata X C18 SPE column (Phenomenex, Torrance, USA), vacuum-dried, reconstituted in 0.5 M TEAB and processed on a TMT6plex kit according to the manufacturer's protocol. The peptide mixtures were then incubated for 2 h at room temperature, pooled, desalted, and dried by vacuum centrifugation.

### Liquid chromatograph-mass spectrometry and database search

The sample was then fractionated by high-pH reverse-phase high- performance liquid chromatography (HPLC) using an Agilent 300Extend C18 column (5 μm particles, 4.6 mm ID, 250 mm length). Briefly, the peptides were first separated into 80 fractions at a gradient from 2 to 60% acetonitrile in ammonium bicarbonate (pH 10, 10 mM) over 80 min. Then the peptides were combined into 18 fractions, dried by vacuum centrifugation, dissolved in 0.1% FA, and then directly loaded onto a reversed-phase pre-column (Acclaim PepMap 100, Thermo Scientific). The peptides were separated using a reversed-phase analytical column (Acclaim PepMap RSLC, Thermo Scientific). The gradient was as follows: first an increase from 7 to 23% solvent B (0.1% FA in 98% ACN) over 26 min, 23 to 35% in 8 min, climbing to 80% in 3 min, and holding at 80% for the last 3 min, all at a constant flow rate of 400 nl/min on an EASY-nLC 1000 UPLC system. The resulting peptides were analyzed by a Q Exactive™ hybrid quadrupole-Orbitrap mass spectrometer (Thermo Fisher Scientific, Waltham, USA).

The peptides were subjected to an NSI source and then tested by MS/MS in Q Exactive™ (Thermo Fisher Scientific) coupled online to the UPLC. Intact peptides were detected in the Orbitrap at a resolution of 70,000. Peptides were selected for MS/MS using NCE of 31, and ion fragments were detected in the Orbitrap at a resolution of 17,500. A data-dependent procedure that alternated between one MS scan and 20 MS/MS scans was applied for the top 20 precursor ions above a threshold ion count of 1E4 in the MS survey scan with 30.0 s dynamic exclusion and at the electrospray voltage of 2.0 kV. Automatic gain control (AGC) was used to prevent overfilling of the ion trap; 5E4 ions were accumulated for generation of MS/MS spectra. For MS scans, the m/z scan range was 350–1800, and the fixed first mass was set as 100 m/z. The acquired data were processed using MaxQuant search engine 1.5.2.8. MS/MS spectra were searched against Swissprot Human concatenated with a reverse decoy database. Trypsin/P was specified as the cleavage enzyme allowing up to 2 missing cleavages. The mass tolerance for precursor ions was set as 20 ppm in First search and 5 ppm in Main search, and that for fragment ions was set as 0.02 Da.

### KEGG pathway analysis

The enriched pathways of the differentially expressed proteins (DEPs) against all identified proteins were detected on the Kyoto Encyclopedia of Genes and Genomes (KEGG) database by a two-tailed Fisher's exact test (http://www.kegg.jp/kegg/ pathway.html) (11). Multiple hypothesis testing was corrected using a standard false discovery rate control method. The significant pathways with corrected *p* < 0.05 were classified into hierarchical categories according to the KEGG website. The quantified proteins were divided into four quantitative categories according to the quantification ratio: CNE2-IR/CNE2 ratio < 0.33; 0.33–0.5; 2–3; > 3 (all *P* < 0.05), which were marked as Q1, Q2, Q3, and Q4, respectively. Then the quantitative category-based clustering was performed. All the substrate categories obtained after enrichment were collated along with their *P*-values, and then filtered to find the categories that were at least enriched in one of the clusters with *P* < 0.01. The filtered *P*-values were transformed by the function x = –log_10_ (*P*-value), then the x values were z-transformed for each category, and finally the z scores were processed by one-way hierarchical clustering (Euclidean distance, average linkage clustering) in Genesis. Cluster membership was visualized by a heat map using the function heatmap.2 from the R-package gplots.

### Protein-protein interaction enrichment analysis

Protein-protein interaction enrichment was analyzed in the gene annotation and analysis resource *Metascape* (http://metascape.org/gp/index.html) (12). The densely connected network components were identified using a molecular complex detection (MCODE) algorithm. Pathway and process enrichment to each MCODE component was analyzed independently and the three best-scoring terms (by *P*-value) were retained as the functional description of the corresponding components. Interaction networks were presented using a graph, where nodes represented proteins and edges represented pair wise interactions. The analysis of the network structure has led to the observation of some apparently recurrent properties of biological networks: power-law degrees distribution, small world, high clustering coefficients, and modularity. The character of small-world means that most nodes in PPIN are not neighbors of one another. Modularity is another characteristic feature of protein-protein interaction (13, 14).

### Clonogenic survival assay

Radiosensitivity was measured by the clonogenic survival assay. Briefly, NPC cells (5 × 10^2^) were plated in 6-cm culture dishes and irradiated (6 Gy). Then after further culture for 12 days, the surviving colonies (defined as a colony with >50 cells) were stained with 0.5% crystal violet and counted.

### Cell viability analysis

The irradiation-caused growth inhibition in NPC cells was detected via the CCK-8 viability assay (15). Briefly, cells (2 × 10^4^/well)were seeded in 24-well culture plates in triplicate for 16 h of incubation and then were irradiated with 6 Gy at room temperature and 300 cGy/min with a linear accelerator (2100EX, Varian, USA). Cell viability was monitored at 48 h after irradiation. Each experiment was repeated four times.

### Western blot

Western blot was conducted as described previously (16). Briefly, cells were lysed and quantified using a bicinchoninic acid protein assay kit (Beyotime Biotechnology, Shanghai, China). Protein lysates (15 μg) were separated by 10% sodium dodecyl sulfate polyacrylamide gel electrophoresis (SDS-PAGE) and transferred to Hybond-P polyvinylidene difluoride (PVDF) membranes (Amersham Biosciences, Uppsala, Sweden). After blocking with 5% non-fat dry milk in a tris-buffered saline buffer for 2 h at room temperature, the blots were incubated at room temperature first with diluted antibodies against various proteins (MAPK15, FYN, IKBKB, MAP2K6, CDK4, dilution 1:1000; γ-H2AX, 1:5000) for 2 h and then with horseradish peroxidase (HRP)-conjugated goat-anti-mouse antibody (Abcam, Cambridge, USA) for 1 h. The signals were visualized with an enhanced chemiluminescence detection reagent (Abcam, Cambridge, USA). β-actin (1:5000) served as a loading control.

### BrdU labeling of cells in proliferative states

The BrdU incorporated into cellular DNA during the S phase was detected with a BrdU cell proliferation assay kit (Cell Signaling, Boston, USA). Briefly, NPC cells were plated into 96-well plates (4 × 10^3^ cells/well) and incubated for 16 h before irradiation. Then at 48 h after 6 Gy irradiation, the NPC cells were labeled with 10 μM BrdU for 2 h and then fixed for DNA denaturing with a fixing/denaturing solution. The incorporated BrdU and the bound detection antibody were detected by adding BrdU mouse antibody and HRP-linked anti-mouse IgG antibody, respectively. HRP substrate TMB was added for color developing, as the magnitude of the absorbance (OD_450_) from the developed color is proportional to the quantity of the incorporated BrdU, which is a direct indication of cells in proliferative states.

### Caspase-3 activity assay

Apoptosis of NPC cells after exposure to irradiation was detected using a caspase-3 colorimetric assay kit (Abcam, Cambridge, USA) based on the cleavage of Asp-Glu-Val-Asp (DEVD)-pNA. Briefly, NPC cells (5 × 10^5^ cells) were ice-lysed for 10 min and centrifuged at 10,000 × g for 1 min. The resulting supernatant in each 96-well flat-bottom microplate was sent to enzyme reactions using 50 μl of cell lysate (100–200 μg of total proteins). The results were expressed as specific activity of caspase-3 (UI/mg protein).

### ROS accumulation measurement

Intracellular ROS was detected by an oxidation-sensitive fluorescent probe—dichlorofluorescin diacetate (DCFDA) as described previously (15). Briefly, NPC cells were plated into 96-well plates (4 × 10^3^ cells/well) and incubated for 16 h before irradiation. At different time points (1, 6, 12 h) after irradiation, NPC cells were treated with 25 μM DCFDA at 37°C for 30 min in the dark, and then subjected to fluorescence reading in an F97Pro fluorospectrometer (Lengguang Technology, Shanghai, China). Florescence DCF defined as an arbitrary unit was read at excitation and emission wavelengths of 485 and 535 nm, respectively.

### Statistical analysis

Statistical analysis was conducted on SPSS 13.0. Single comparisons were performed using Student's *t*-test or Mann–Whitney's U test. All statistical tests were two-sided. Differences at *p* < 0.05 were considered statistically significant.

## Results

### Quantitative proteomic analysis on CNE2 and CNE2-IR

**Figure 1:**
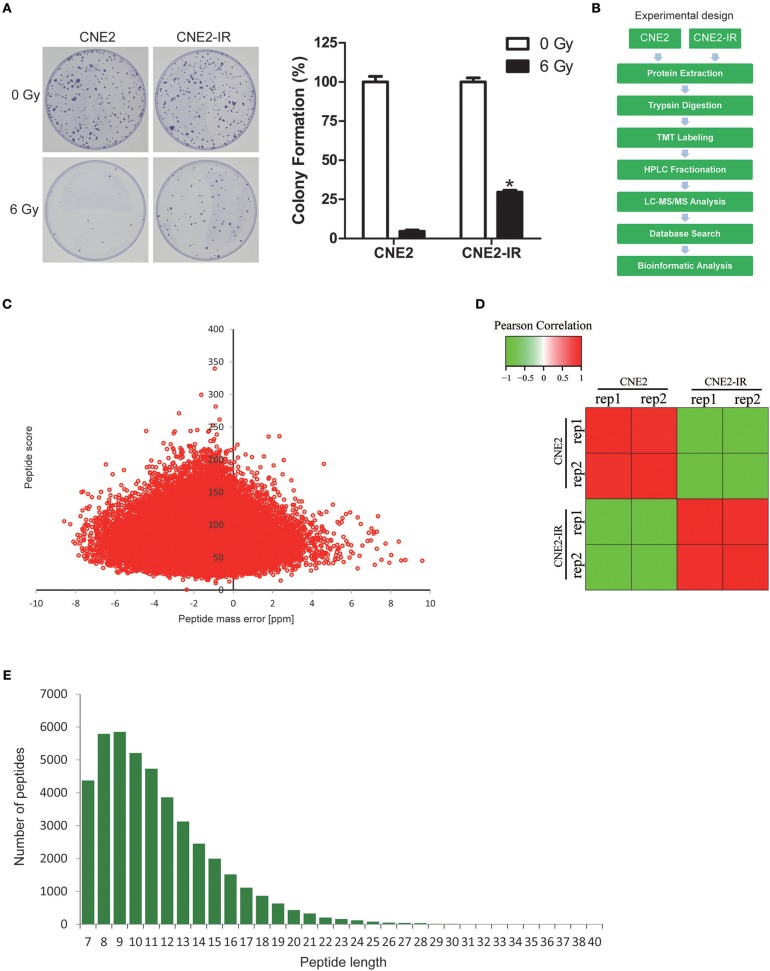
Quantitative proteomic analysis on CNE2 and its radioresistant subline CNE2-IR. **(A)** Clonogenic survival analysis on the radiosensitivity of CNE2 and CNE2-IR. The colony formation in CNE2 or CNE2-IR without ionizing irradiation was regarded as 100%, respectively. CNE2-IR 6 Gy vs. CNE2 6 Gy, *P < 0.05. **(B)** Experimental scheme for the quantitative proteomic analysis on CNE2 and CNE2-IR. **(C)** QC validation of MS data. Mass error indicates distribution of all identified peptides. **(D)** Reproducibility of the quantitative proteomic analysis on CNE2 and CNE2-IR. **(E)** Peptide length distribution identified by quantitative proteomic analysis.

NPC cell line CNE2 and its radioresistant subline CNE2-IR were previously tested by 2-DE/MALDI-TOF-MS to identify potential biomarkers for predicting radiosensitivity in NPC cells (9, 17). To verify the radioresistant phenotypes of CNE2-IR, CNE2-IR, and its parental CNE2 cells were irradiated with a 6 Gy dose and examined by clonogenic survival assay. Consistently, CNE2-IR showed much higher clonogenic potential than CNE2 after exposure to irradiation (Figure 1A). Hence, these two cell lines were used for quantitative proteomic analysis using TMT labeling and LC-MS/MS. The general experimental strategy was illustrated in Figure 1B. Totally 5,946 protein groups were identified, among which 5,185 proteins were quantified (Table S1). Firstly, mass error analysis showed the mass errors of all the identified peptides were near zero and mostly < 10 ppm, suggesting the mass accuracy of the MS data meets the requirement (Figure 1C). The protein detection and quantification showed high reproducibility (Figure 1D). Secondly, the length of most peptides was between 8 and 16, which agrees with the property of tryptic peptides (Figure 1E). Several radioresistance-related proteins [e.g., superoxide dismutase 2 (SOD2), heat shock protein 1 (HSPB1), peroxiredoxin 1 (PRDX1), nucleoside diphosphate kinase 1 (NME1), nucleophosmin 1 (NPM1)] identified in previous 2-DE/MALDI-TOF-MS studies in a CNE2-IR/CNE2 cell model were also observed in our dataset (Table S2).

**Figure 2:**
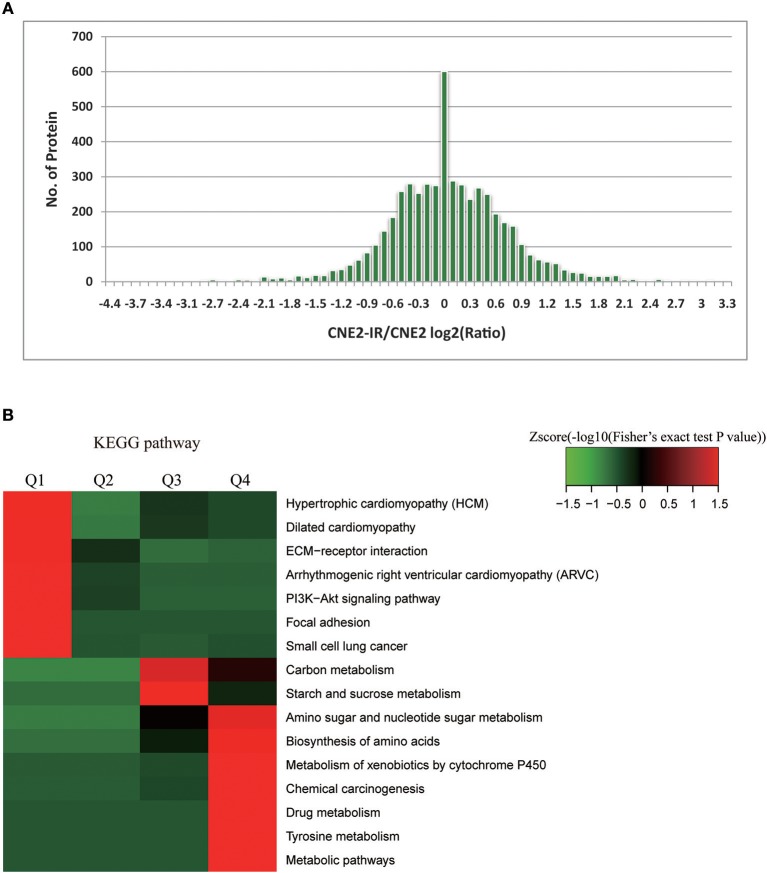
Bioinformatics analysis on radiosensitivity-related proteome in CNE2 and CNE2-IR. **(A)** Enrichment of differentially expressed proteins in CNE2 and CNE2-IR. Among the 754 differentially expressed proteins, 440 proteins were with increased level (CNE2-IR/CNE2, fold change ≥2) and 314 with decreased level (CNE2-IR/CNE2, fold change ≤ 0.5). **(B)** Intensive bioinformatic analysis on 754 differentially expressed proteins based on KEGG pathway analysis. The quantifiable proteins in this study were divided into four quantitative categories according to the CNE2-IR/CNE2 Ratio: Q1 (CNE2-IR/CNE2 Ratio < 0.33 and *P*-value < 0.05), Q2 (0.33 < CNE2-IR/CNE2 Ratio < 0.5 and *P*-value < 0.05), Q3 (2 < CNE2-IR/CNE2 Ratio < 3 and *P*-value < 0.05) and Q4 (CNE2-IR/CNE2 Ratio >3 and *P*-value < 0.05).

Among the 5,185 quantifiable proteins, 440 proteins were upregulated and 314 downregulated, with CNE2-IR/CNE2 fold change ≥2 and ≤ 0.5, respectively (Figure 2A, Table S3). Intensive bioinformatics for 754 DEPs was finally analyzed. Radiosensitivity-related cellular pathways and protein complex were identified through the clustering analysis based on KEGG pathways (Figure 2B). The quantifiable proteins were divided into four quantitative categories as stated above. Results showed the dominant pathways enriched in the increased proteins (Q4) in CNE2-IR cells were the pathways of amino sugar/nucleotide sugar metabolism, amino acid biosynthesis, xenobiotics metabolism by cytochrome P450/drug metabolism, tyrosine metabolism, and metabolic pathway such as glycolysis.

**Figure 3:**
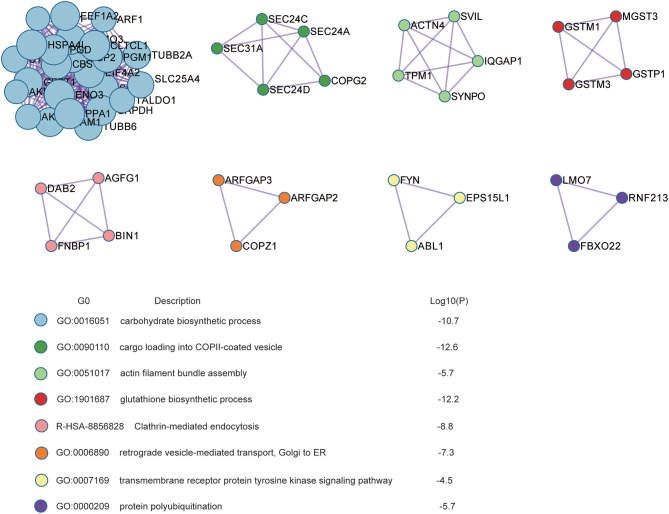
Protein interaction networks based on Protein-Protein interaction enrichment analysis on radioresistance-related 440 proteins.

Protein-protein interaction enrichment analysis was conducted to explore the protein interaction networks encoded by all 440 radioresistance-related proteins (Figure 3). In the networks, radioresistance-related proteins usually interact to form a larger network. Gene Ontology (GO) analysis suggests these proteins have important functions in carbohydrate biosynthesis, vesicle trafficking, actin filament bundle assembly, glutathione synthesis, clathrin-mediated endocytosis, receptor tyrosine kinase signaling, and protein polyubiquitination. Our findings might depict a complicated network that integrates multiple pathways/processes involved in the radioresistance development of NPC cells.

### MAPK15 might be involved in the radioresistance regulation in NPC cells

**Figure 4:**
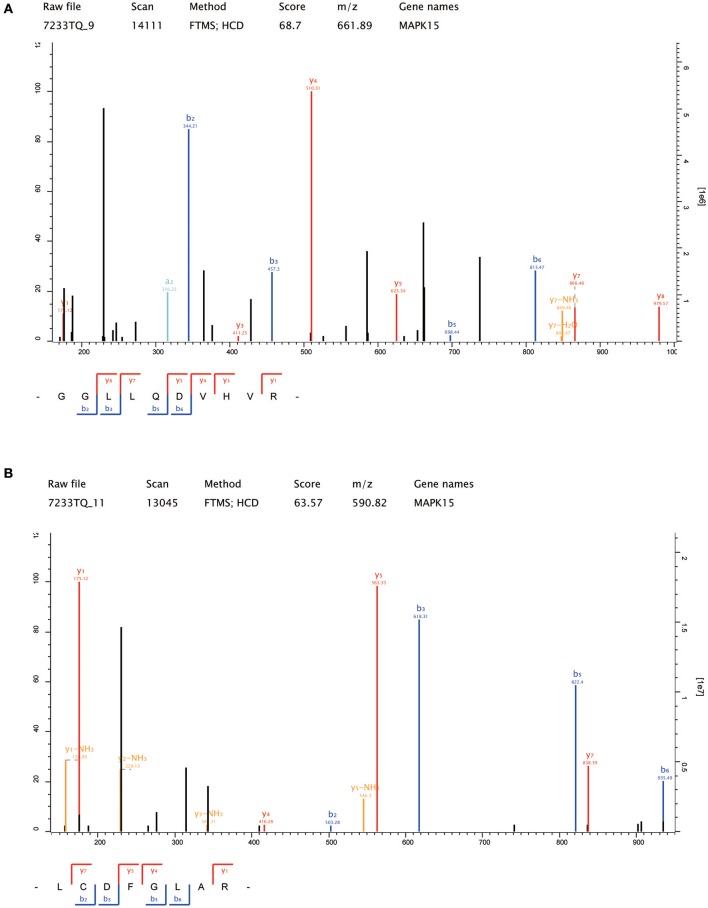
The amino acid sequences of the two unique MAPK15 peptides identified by LC-MS/MS. **(A)** GGLLQDVHVR; **(B)** LCDFGLAR.

Thirteen up-regulated protein kinases in CNE2-IR cells were screened out according to the following criteria: fold change ≥2 (CNE2-IR/CNE2) and at least two unique peptides identified. The details of the identified protein kinases were summarized in Table 1. Several protein kinases [IKBKB, MAP2K6, TANK binding kinase 1 (TBK1), Rho-associated coiled-Coil containing protein kinase (ROCK2), TAO Kinase 3, CDK4, serine/threonine kinase (STK38), CDK5, non-receptor tyrosine kinase (ABL1), and checkpoint kinase (CHEK2)] were reportedly closely related to radioresistance in various cancers, while MAPK15, FYN, and WNK lysine-deficient protein kinase 1 (WNK1) still await further study on their functions in the radioresistance development of cancer cells. Notably, MAPK15 (also known as ERK8) was shown to be the most enriched protein kinase in CNE2-IR cells (CNE2-IR/CNE2, fold change >7). The amino acid sequences of the two unique MAPK15 peptides were identified as GGLLQDVHVR and LCDFGLAR (Figures 4A,B). Therefore, MAPK15 was selected as a potential target for further investigation.

**Table 1:** Radioresistance-related proteins in CNE2-IR cells.

**Protein accession**	**Gene name**	**CNE2IR/CNE2 Ratio**	**Regulated Type**	**CNE2IR/CNE2 *P* value**	**Score**	**Coverage [%]**	**Peptides**	**PSMs**	**Unique peptides**
Q8TD08	MAPK15	7.375	Up	0.000542	59.831	3.3	2	3	2
P06241	FYN	4.594	Up	0.00224	60.761	6.7	4	4	2
O14920	IKBKB	3.017	Up	0.00232	86.563	7.9	5	6	5
P52564	MAP2K6	2.953	Up	0.00646	37.492	13.8	5	6	2
P11802	CDK4	2.944	Up	0.00596	33.729	19.5	5	5	4
O75116	ROCK2	2.544	Up	0.000197	160.9	16.2	22	24	19
Q9H2K8	TAOK3	2.459	Up	0.00206	105.4	7.9	7	9	4
Q9H4A3	WNK1	2.444	Up	0.00424	250.79	4.3	9	13	9
Q15208	STK38	2.416	Up	0.0271	54.19	6.2	3	3	3
Q00535	CDK5	2.163	Up	0.00116	33.304	26	7	8	6
Q9UHD2	TBK1	2.136	Up	0.0458	83.641	13.4	8	8	8
P00519	ABL1	2.055	Up	0.0102	122.87	2.8	2	2	2
O96017	CHEK2	2.034	Up	0.00338	60.914	4.2	2	2	2

### Knockdown of MAPK15 attenuated radioresistance in CNE2-IR cells

**Figure 5:**
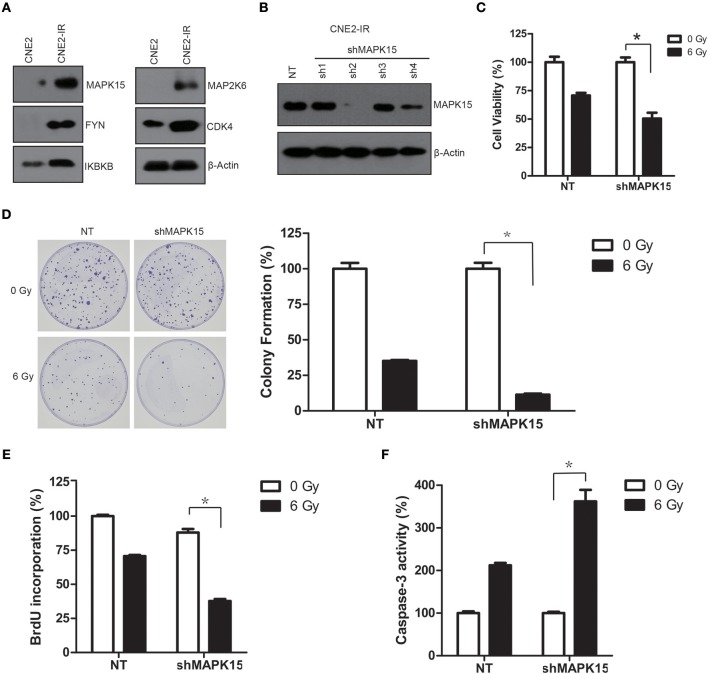
High protein levels of MAPK15 regulate the radiosensitivity in CNE2-IR cells. **(A)** Western blotting validation on protein levels of five protein kinases (MAPK15, FYN, IKBKB, MAP2K6, and CDK4) in CNE2 and CNE2-IR cells. β-actin served as a loading control. **(B)** MAPK15 protein expression was significantly reduced in CNE2-IR cells transduced with shMAPK15 lentivirus (sh4). **(C)** Knockdown of MAPK15 increased radiosensitivity in CNE2-IR cells. The cell viability in NT or shMAPK15 without ionizing irradiation was regarded as 100%, respectively. shMAPK15 (6 Gy) vs. NT (6 Gy), *n* = 3 *P < 0.05. **(D)** Knockdown of MAPK15 attenuated colony formation after irradiation in CNE2-IR cells. The colony formation in NT or shMAPK15 without ionizing irradiation was regarded as 100%, respectively. shMAPK15 (6 Gy) vs. NT (6 Gy), *n* = 3, *P < 0.05. **(E)** Knockdown of MAPK15 impaired the proliferative potential of CNE2-IR cells exposed to irradiation. The BrdU incorporation in NT without ionizing irradiation was regarded as 100%. shMAPK15 (6 Gy) vs. NT (6 Gy), *n* = 4, *P < 0.05. **(F)** Knockdown of MAPK15 increased caspase-3 activity following irradiation in CNE2-IR cells. The caspase-3 activity in NT or shMAPK15 without ionizing irradiation was regarded as 100%, respectively. shMAPK15 (6 Gy) vs. NT (6 Gy), *n* = 4, *P < 0.05.

To confirm the expressions of the differential protein kinases identified by proteomics, the expressions of MAPK15, FYN, IKBKB, MAP2K6, and CDK4 in the CNE2-IR and CNE2 cells were detected by Western blot. Results showed these five protein kinases were all significantly more expressed in CNE2-IR than in CNE2 (Figure 5A), which were consistent with the proteomic analysis.

To address whether the MAPK15 expression level may affect the radiosensitivity of CNE2-IR, CNE2-IR cells were transduced with non-targeting (NT) or specific shRNA targeting MAPK15 (shMAPK15), and cell radiosensitivity was determined by clonogenic survival, cell viability, caspase-3 colorimetric, and cell cycle assays. MAPK15 expression was significantly reduced in the CNE2-IR cells transduced with shMAPK15 lentivirus (sh4), but was not significantly affected by NT shRNA compared with CNE2 cells (Figure 5B). Therefore, shRNA sh2 was used to attenuate the endogenous MAPK15 expression. Then the effect of MAPK15 expression on the radiosensitivity of CNE2-IR cells was evaluated by cell viability assay, which showed shMAPK15 transduction significantly intensified the radiosensitivity of CNE2-IR cells compared with NT shRNA (*P* < 0.05; Figure 5C). Furthermore, colony formation of CNE2-IR cells in the shMAPK15 group decreased 48 h after 6 Gy irradiation compared with the NT shRNA group (*P* < 0.05; Figure 5D). The function of MAPK15 was further studied by BrdU labeling assay. At 48 h after 6 Gy irradiation, fewer CNE2-IR cells in the shMAPK15 group were in the proliferative state compared with the CNE2-IR NT group (Figure 5E). Consistently, more caspase-3 activity was detected in the CNE2-IR shMAPK15 group than in the CNE2-IR NT shRNA group at 48 h after 6 Gy irradiation (*P* < 0.05; Figure 5F). These results suggest MAPK15 might be an important regulator of radioresistance in CNE2-IR cells.

### Over-expression of MAPK15 promotes radioresistance in NPC cell lines

**Figure 6:**
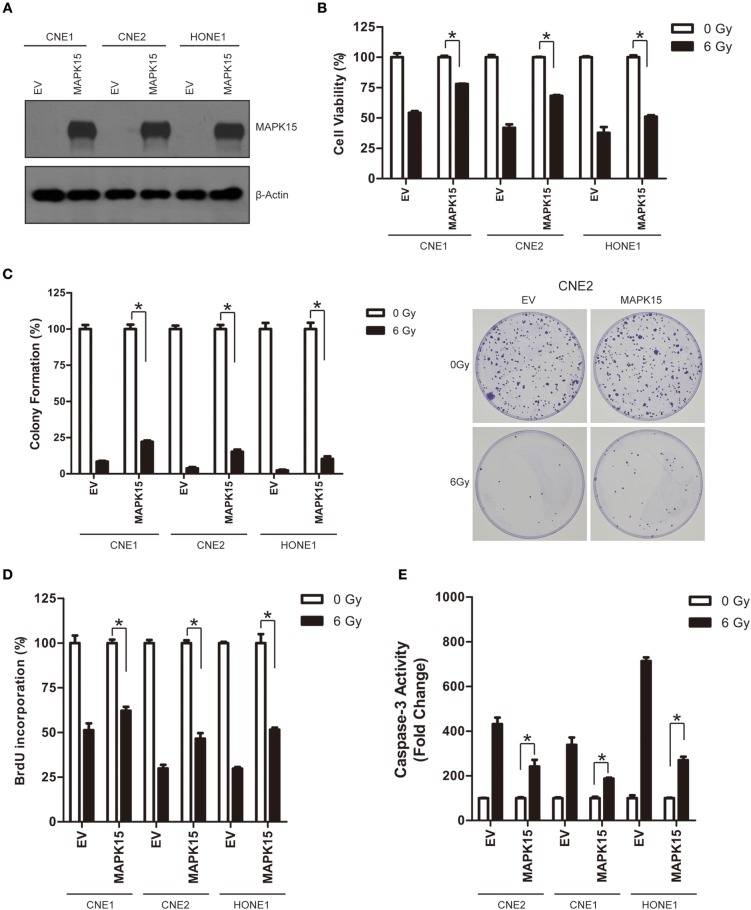
Over-expression of MAPK15 promotes radioresistance in NPC cells. **(A)** Transfection of MAPK15 plasmid considerably increased MAPK15 protein expression in three NPC cell lines CNE1, CNE2, and HONE1. **(B)** Over-expression of MAPK15 increased radioresistance in NPC cells. The cell viability in vector control (EV) or MAPK15 without ionizing irradiation was regarded as 100%, respectively. MAPK15 (6 Gy) vs. EV (6 Gy), *n* = 3, *P < 0.05. **(C)** Over-expression of MAPK15 increased colony formation after irradiation in NPC cells. The colony formation in EV or MAPK15 without ionizing irradiation was regarded as 100%, respectively. MAPK15 (6 Gy) vs. EV (6 Gy), *n* = 3, *P < 0.05. **(D)** Over-expression of MAPK15 enhanced the proliferative potential of NPC cells exposed to irradiation. The BrdU incorporation in EV or MAPK15 without ionizing irradiation was regarded as 100%. MAPK15 (6 Gy) vs. EV (6 Gy), *n* = 4, *P < 0.05. **(E)** Over-expression of MAPK15 decreased caspase-3 activity following irradiation in NPC cells. The caspase-3 activity in EV or MAPK15 without ionizing irradiation was regarded as 100%, respectively. MAPK15 (6 Gy) vs. EV (6 Gy), *P < 0.05.

We also constitutively tested MAPK15 expression in three NPC cell lines (CNE1, CNE2, and HONE1) to confirm whether MAPK15 could alter the radiosensitivity of NPC cells. NPC cell lines were transfected with a plasmid construct expressing empty vector control (EV) or MAPK15, and then cell radiosensitivity was determined by clonogenic survival, caspase-3 colorimetric, cell cycle, and cell viability assays. MAPK15 expression was significantly increased in CNE1, CNE2, and HONE1 cells (Figure 6A), whereas it was not significantly altered by the vector control. We next evaluated the effect of MAPK15 expression on the radiosensitivity of NPC cells by cell viability assay. Results showed higher cell viability in the MAPK15 group at 48 h after 6 Gy irradiation (Figure 6B). Furthermore, the colony formation of MAPK15-expressing NPC cells after 6 Gy irradiation increased compared with the vector control (Figure 6C).

At 48 h after 6 Gy irradiation, more NPC cells in the MAPK15 group were in proliferative states compared with the vector control (Figure 6D). Consistently, less caspase-3 activity was detected in MAPK15-expressing NPC cells than in the vector control at 48 h after irradiation (*P* < 0.05; Figure 6E). The effect of MAPK15 on BrdU incorporation was further analyzed, which showed MAPK15 might be involved in the radioresistance regulation of NPC cells.

### MAPK15 regulates ROS accumulation and DNA damage repair in NPC cells

**Figure 7:**
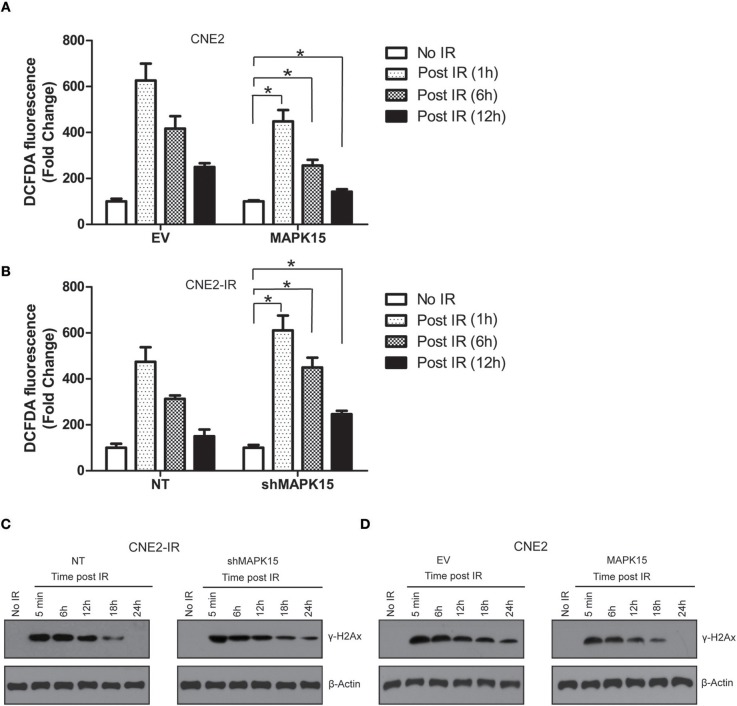
MAPK15 regulates ROS accumulation and DNA damage repair in NPC cells. **(A)** Over-expression of MAPK15 enhanced the neutralization of ROS accumulation in CNE2 cells exposed to irradiation. The DCFDA fluorescence in EV or MAPK15 without ionizing irradiation was regarded as 100%. MAPK15 (1 h, 6 h, 12 h) vs. EV (1 h, 6 h, 12 h), *n* = 4, *P < 0.05, respectively. **(B)** Knockdown of MAPK15 delayed the attenuation of ROS in CNE2-IR cells exposed to irradiation. The DCFDA fluorescence in NT or shMAPK15 without ionizing irradiation was regarded as 100%. shMAPK15 (1 h, 6 h, 12 h) vs. NT (1 h, 6 h, 12 h), *n* = 4, *P < 0.05, respectively. **(C)** Knockdown of MAPK15 delayed the DNA damage repair in CNE2-IR cells exposed to irradiation. γ-H2AX was used as a marker to reflect the double strand DNA breaks (DSBs) that are produced by ionizing radiation. β-actin served as a loading control. **(D)** Over-expression of MAPK15 in CNE2 cells promoted the DNA damage repair in CNE2-IR cells exposed to irradiation.

Irradiation generates a large quantity of ROS, which is required for DNA damage, cell cycle redistribution, apoptosis, and cytotoxicity (16). We further studied the effects of MAPK15 on ROS accumulation and DNA damage repair in the irradiated NPC cells. ROS accumulation after exposure to irradiation (6 Gy) was detected in NPC cells expressing the vector control or MAPK15. Less ROS was observed in the MAPK15-expressing NPC cells than in the vector control at different time points (1, 6, 12 h) in all the three NPC cell lines (Figure 7A), while knockdown of MAPK15 led to higher ROS accumulation compared to the NT control in the irradiated CNE2-IR cells (Figure 7B). The histone variant H2AX is rapidly phosphorylated (γ-H2AX) in large chromatin domains (foci) flanking double strand DNA breaks (DSBs) that are produced by ionizing radiation. We compared the γ-H2AX formation and elimination dynamics after γ-irradiation between CNE2 cells with or without MAPK15 expression. The γ-H2AX levels in CNE2-IR cells were analyzed by Western blotting at 5 min, 6, 12, 18 and 24 h post-irradiation. At 5 min, the γ-H2AX level in MAPK15-expressing cells was comparable to the vector control group (Figure 7C). However, after 18 h of recovery, the γ-H2AX level in the MAPK15-expressing cells was significantly reduced from the 5 min time point, whereas γ-H2AX level was still considerable in the shMAPK15-expressing cells. Then MAPK15 was over-expressed in CNE2 cells to verify the role of MAPK15 in DNA repair of DSBs. Over-expression of MAPK15 led to rapid decline of γ-H2Ax level post-irradiation compared to the vector control group (Figure 7D). Therefore, the increased MAPK15 level enhanced DNA repair of DSBs, as assayed by the biomarker γ-H2AX.

## Discussion

Radioresistance remains a major problem in NPC treatment (18, 19), but the molecular mechanisms are still poorly understood. Quantitative proteomic techniques have been widely applied in preclinical and clinical investigations due to their ability to reveal the dynamics of protein expression and protein-protein interactions from a global perspective, which greatly help to understand the gene function in cellular processes.

The proteomic approach has been introduced into the research field to identify radioresistance-associated DEPs. Two 2-DE and MALDI-TOF-MS studies based on the CNE2/CNE2-IR system identified 16 DEPs in Li et al. and 34 DEPs in Feng et al. (9, 17). Herein, quantitative TMT labeling and LC-MS/MS were used to screen out the DEPs in the CNE2/CNE2-IR system. The detection and quantification identified 5,946 protein groups and quantified 5,185 proteins. Compared with the previous two studies, our quantitative proteomic analysis successfully identified 754 DEPs (fold change ≥ 2), which highlights the high sensitivity and reproducibility of this technique in identifying radiosensitivity-related proteins. The DEPs identified here could further be classified into various metabolic processes (e.g., amino sugar and nucleotide sugar metabolism, biosynthesis of amino acids, drug metabolism, tyrosine metabolism, and metabolic pathway such as glycolysis). Protein-protein interaction analysis also revealed multiple biological processes and pathways were activated in radioresistant CNE2-IR cells. Therefore, our findings would depict these important metabolic process/signaling networks that were potentially involved in the radioresistance of NPC cells.

Protein kinases are important in carcinogenesis and are prioritized as drug targets. Our comparative proteomic analysis identified thirteen potential radioresistance- associated protein kinases in NPC cells. MAPK15 was selected for further functional study owing to its highest enrichment in radioresistant CNE2-IR cells. Functional abnormality of MAPK15 is frequently seen in cancer initiation and progression. As an important cancer-related MAP kinase, MAPK15 regulates cellular function in cancer cells through multiple mechanisms. MAPK15 is highly expressed in human lung cancer cell lines and promotes the activation of NF-κB (20). MAPK15 is a novel HuR kinase that regulates tumor suppressor PDCD4 through a miR-21 dependent mechanism (21). MAPK15 also mediates BCR-ABL1- induced autophagy and regulates oncogene-dependent cell proliferation and tumor formation in human chronic myeloid leukemia (22). Over-expression of MAPK15 is associated with copy number gain and contributes to the c-Jun stability in gastric cancer (23). MAPK15-mediated c-Jun phosphorylation increases tumorigenesis of human colon cancer (24).

MAPK15 might be an important regulator of radioresistance in NPC. Our findings showed depletion of MAPK15 expression decreased clonogenic survival, cell viability, and increased cell apoptosis in irradiated CNE2-IR cells, while over-expression of MAPK15 promoted cell survival and decreased apoptosis in NPC cell lines. Although some studies have linked the molecular function of MAPK15 to DNA damage repair, the role of MAPK15 in radioresistance regulation still awaits further investigation. As reported, MAPK15 could be induced and activated by hydrogen peroxide, DNA alkylating and cross-linking agents and poly-(ADP-ribose) polymerase inhibitor (25). Similarly, we found MAPK15 expression was highly enriched in NPC cells with induced resistance to ionizing irradiation. The up-regulation of MAPK15 reportedly prevents DNA damage in male germ cell tumors (26). As a chromatin-bound kinase, MAPK15 could protect genomic integrity by inhibiting HDM2-mediated degradation of the DNA clamp PCNA (27). Consistently, we found MAPK15 could promote radiotherapy-induced DNA damage repair in NPC cells. Radiotherapy can cause DNA breaks and induce cell apoptosis by inducing ROS. Cancer cells that possess more abundant endogenous antioxidant systems are typically more radioresistant (28). We also found MAPK15 could attenuate radiotherapy-induced ROS accumulation, although the detailed mechanisms are still poorly understood. We speculate that MAPK15 may affect the NADPH oxidases that are the key enzymes of redox signaling and the main source of ROS *in vivo*. More importantly, NADPH is very important for glycolysis in tumor cells, and its inhibition could significantly inhibit tumor growth. Overexpression of MAPK15 may diminish the activity of NADPH and the release of ROS in radiated NPC cells. Nevertheless, further study is needed.

To the best of our knowledge, this is the first study reporting that MAPK15 over-expression is associated with the induced radioresistance. Furthermore, MAPK15 might regulate radioresistance through attenuating ROS accumulation and promoting DNA damage repair in irradiated NPC cells. MAPK15 may be a novel target or radioresistance in NPC cells. Future study should focus on three aspects. (I) Clinical samples should be collected to validate the MAPK15 expression in NPC patients. (II) We previously identified MAP2K6 as a potential regulator of LIFR- induced radioresistance in NPC cells (29), but the protein-protein interaction or up- and down-stream regulation should be considered in mechanism analysis, such as FYN. (III) Animal experiments are needed for further confirmation, and relevant inhibitor should be developed to observe the inhibition effect of MAPK15 on radiation in NPC cells and patients.

In summary, the quantitative proteomic approach was used to identify a radioresistance-related protein profile in NPC. MAPK15 contributes to NPC radioresistance and could be a novel potential biomarker for predicting the response of NPC cells to radiotherapy. MAPK15 might potentially regulate radioresistance through attenuating ROS accumulation and enhancing DNA damage repair in NPC cells. Targeting MAPK15 might be useful in sensitizing NPC cells to radiotherapy. The findings could have clinical value in distinguishing radiosensitive from radioresistant NPC and in identifying subgroups of NPC patients who could benefit from personalized therapeutic strategies.

## Data availability statement

All datasets for this study are included in the manuscript and the supplementary files.

## Author contributions

LS and JF designed the study. ZL and NL performed the experiments. LS and ZL analyzed the data. JF wrote the manuscript. All authors have read and approved the final version of the manuscript.

### Conflict of interest statement

The authors declare that the research was conducted in the absence of any commercial or financial relationships that could be construed as a potential conflict of interest.

## Supplementary material

Table S1 Quantitative proteomic analysis using TMT labeling and LC-MS/MS analysis. In total, 5,946 protein groups were identified, among which 5,185 proteins were quantified.

Table S2 Radiosensitivity-related proteins identified in previous 2-DE/ MALDI-TOF-MS studies and in our study.

Table S3 Intensive bioinformatic analysis identified 754 differentially expressed proteins with fold change ≥2.
